# Differences in cerebral response to esophageal acid stimuli and psychological anticipation in GERD subtypes - An fMRI study

**DOI:** 10.1186/1471-230X-11-28

**Published:** 2011-03-26

**Authors:** Kun Wang, Li-Ping Duan, Xiang-Zhu Zeng, Jian-Yu Liu, Weng Xu-Chu

**Affiliations:** 1 Department of Gastroenterology, Peking University Third Hospital, Beijing, 100191, P.R. China; 2 Department of Radiology, Peking University Third Hospital, Beijing, 100191, P.R. China; 3 Institute of Psychology, Chinese Academy of Sciences, Beijing, 100101, P.R. China

## Abstract

**Background:**

To evaluate whether there are differences in the cerebral response to intraesophageal acid and psychological anticipation stimuli among subtypes of gastroesophageal reflux disease (GERD).

**Methods:**

Thirty nine patients with GERD and 11 healthy controls were enrolled in this study after gastroscopy and 24 hr pH monitoring. GERD subjects were divided into four subgroups: RE (reflux esophagitis), NERD+ (non-erosive reflux disease with excessive acid reflux), NERD-SI+ (normal acid exposure and positive symptom index) and NERD-SI+ (normal acid exposure and negative symptom index, but responded to proton pump inhibitor trial). Cerebral responses to intraesophageal acid and psychological anticipation were evaluated with fMRI.

**Results:**

During intraesophageal acid stimulation, the prefrontal cortex (PFC) region was significantly activated in all subgroups of GERD; the insular cortex (IC) region was also activated in RE, NERD+ and NERD-SI- groups; the anterior cingulated cortex (ACC) region was activated only in RE and NERD-SI- groups. The RE subgroup had the shortest peak time in the PFC region after acid was infused, and presented the greatest change in fMRI signals in the PFC and ACC region (*P *= 0.008 and *P *= 0.001, respectively). During psychological anticipation, the PFC was significantly activated in both the control and GERD groups. Activation of the IC region was found in the RE, NERD-SI+ and NERD-SI- subgroups. The ACC was activated only in the NERD-SI+ and NERD-SI- subgroups. In the PFC region, the NERD-SI- subgroup had the shortest onset time (*P *= 0.008) and peak time (*P *< 0.001). Compared with actual acid infusion, ACC in RE and IC in NERD+ were deactivated while additional areas including the IC and ACC were activated in the NERD-SI+ group; and in NERD-SI- group, onset-time and peak time in the PFC and IC areas were obviously shorter in induced anticipation than in actual acid infusion.

**Conclusions:**

The four subgroups of GERD patients and controls showed distinctly different activation patterns and we therefore conclude GERD patients have different patterns of visceral perception and psychological anticipation. Psychological factors play a more important role in NERD-SI+ and NERD-SI- groups than in RE and NERD+ groups.

## Background

Gastroesophageal reflux disease (GERD) is a common disorder which is complex. It is defined as a condition that develops when reflux of stomach contents causes troublesome symptoms and/or complications [1]. The pathogenesis of GERD as an entity is diverse. In addition to acid reflux and motor dysfunction, visceral hypersensitivity and psychological factors appear to be important mechanisms of symptom generation in gastroesophageal reflux [2,3].

Reflux esophagitis (RE) with mucosal erosion or ulcer formation, and non-erosive reflux disease (NERD) without overt evidence of mucosal abnormality are the two main phenotypes of GERD. An estimated 50% to 70% of GERD is NERD [4,5]. NERD is considered to be a heterogeneous group because of the different acid reflux characteristics and symptom patterns which it may display. NERD can be divided into three subgroups which include NERD+ with excessive acid reflux, NERD-SI+ with normal acid exposure and a positive symptom index (SI), and NERD-SI- with normal acid exposure and a negative symptom index [6]. To differentiate NERD-SI- and functional heartburn, the Rome III Committee for Functional Esophageal Disorders redefined functional heartburn, and consequently redefined NERD, primarily by placing the hypersensitive esophagus group and those patients with negative symptom association who are responsive to proton pump inhibitor (PPI) treatment in the NERD group [7,8].

Visceral hypersensitivity has been demonstrated in GERD patients. Rodriguez-Stanley et al suggested esophageal hypersensitivity may be a major cause of heartburn [9]. Fass et al performed a modified acid perfusion test in GERD patients and confirmed the presence of acid hypersensitivity [10]. Several studies have also reported acid exposure can enhance esophageal mechanosensitivity in healthy individuals [11-14]. In response to acid exposure cerebral activity occurs more rapidly and with greater intensity in GERD patients than in healthy controls [15].

On the other hand, psychological factors also play a role in GERD. A population-based study showed that psychological scores for neuroticism, anxiety and depression were higher in GERD patients than those in healthy controls [16,17]. Moreover, psychological disorders were found to be positively correlated with heartburn symptoms [18]. Psychological distress may even influence the outcome of laparoscopic Nissen fundoplication in GERD patients [19]. Further studies have suggested that psychological states may modulate esophageal sensitivity in GERD patients through both peripheral and central mechanisms [20,21].

The two subtypes of GERD known as RE and NERD has been reported to have differing epidemiological features and different responses to treatment. Thus, differences in the pathogenesis of RE and NERD are to be expected. In addition, NERD patients have been divided into three subtypes based on clinical manifestations, and particularly on acid reflux characteristics. However, whether there are differences in the pathogenesis among these three subtypes of NERD is still in question. There have been conflicting results regarding visceral sensitivity in RE versus NERD. Wu et al found NERD had a higher positive ratio in the acid perfusion test than RE and suggested NERD characteristically shows higher esophageal acid hypersensitivity [22]. In contrast Hong et al suggested that no difference exists between visceral hypersensitivity in patients with NERD and those with erosive esophagitis [23]. Similarly conflicting results have been reported regarding the role of psychological factors in RE and NERD. Ang et al demonstrated a significantly higher prevalence of minor psychiatric co-morbidity in NERD patients (46.7%) as compared to those with RE (26.4%). In contrast, Xu et al reported no differences in psychiatric scores in RE and NERD [24]. Fass et al suggested there were no differences in perceived stress and autonomic response in patients with RE and NERD. However, to our knowledge, there have been no previous studies of this type which have assessed the effects of visceral stimulation and psychological anticipation in the three subtypes of NERD (NERD+, NERD-SI+ and NERD-SI-) and RE.

fMRI may be used to obtain patient cerebral activation data. Several different cerebral regions including the sensory/motor, parieto-occipital region, prefrontal cortex (PFC), anterior cingulate cortex (ACC), insular cortex (IC) and cerebellum have been reported to participate in the cerebral processing of visceral afferent signals. The PFC, ACC and IC in particular have been reported to participate in esophageal hypersensitivity. In addition, researchers have reported on stimulation patterns in an esophageal sensitivity study [25-29], and visceral pain anticipation studies have also been carried out in healthy controls and irritable bowel disease (IBS) patients [29,30].

The aim of our study was to evaluate whether there are differences in cerebral response to esophageal acid and psychological anticipation stimuli among the four subtypes of GERD and healthy controls by use of fMRI, and to further analyze for potential differences in visceral sensitivity and psychological factors in NERD+, NERD-SI+, NERD-SI- and RE.

## Methods

### Ethics

This study was approved by the ethical committee of Peking University Health Science Center (reference number 0565), and all subjects gave informed consent in writing before commencement of the study.

### Subjects

We randomly enrolled 44 right-handed GERD patients who exhibited typical GER symptoms of heartburn and acid regurgitation at least twice a week together with 12 healthy controls. Among these, 5 patients and 1 control did not complete the study due to failure in cooperating with the testing sequence. The remaining 39 patients and 11 controls completed the protocol. After gastroscopy, ambulatory 24-hr esophageal pH monitoring and PPI trials, GERD patients were divided into 9 cases of RE (7 males/2 females, 56.7 ± 5.9 yrs), 11 cases of NERD+ (6 males/5 females, 44.5 ± 3.9 yrs), 8 cases of NERD-SI+ (4 males/4 females, 58.1 ± 3.8 yrs), and 11 cases of NERD-SI- (5 males/6 females, 47.9 ± 2.2 yrs). Criteria for exclusion from the study included such diseases as peptic ulcer, digestive cancer, previous abdominal surgery, Barrett's esophagus, IBS, diabetes mellitus, and the use of sedatives, selective serotonin reuptake inhibitors or other medication that might affect symptom perception. The patients who had taken PPIs during the previous 4 weeks were also excluded. The 11 healthy volunteers (5 males/6 females, 38.0 ± 3.7 yrs) were enrolled as controls after it was determined they had no gastrointestinal disorders through assessment of health history, reflux diagnostic questionnaires (RDQ), endoscopy and 24-hr pH monitoring.

### Protocol

All the patients and controls completed a RDQ and Symptom Check List-90 (SCL-90) psychological questionnaire, followed by gastroscopy and ambulatory 24-hr pH monitoring. The concept of GERD and general pathogenesis of acid reflux causing heartburn was explained to all subjects. Then they underwent an fMRI study.

### GERD symptom assessment

GERD symptoms were evaluated with the RDQ, which includes two sections to assess the frequency and extent of symptoms including heartburn, acid regurgitation, food reflux and chest pain. These two sections have a total of 24 points. When the subject's score is ≥12, he is considered to have GERD. Patients were required to complete the questionnaire based on their symptoms over the preceding four weeks.

### Assessment of esophageal mucosa

All subjects underwent gastroscopy (Olympus GIF) after fasting overnight. The esophagus was carefully evaluated for presence of mucosal injury. The extent of the esophageal mucosal damage was assessed using the Los Angeles grading system. The stomach and duodenum were also inspected to exclude possible lesions. Routine biopsies were taken in the gastric antrum and duodenal bulb to exclude eosinophilic gastroenteritis.

### Acid reflux quantification and PPI trials

The extent of esophageal acid exposure was determined using the ambulatory Digitrapper MK III pH monitoring system (Synectic Medical, LTD, Sweden). After fasting overnight, a catheter with two pH probes was inserted via the nose into the esophagus; the proximal pH sensor was placed 5 cm above the upper limit of the lower esophageal sphincter (LES). Patients were asked to record their daily activities. Excess esophageal acid exposure was defined as pH < 4 over more than 5% of the total recording time [31], and analysis of recorded data was performed using standard commercially available software. Patients with pathological acid reflux but without esophagitis were classified as NERD+. Individuals without pathologic acid reflux and without esophagitis were classified as NERD-. Subsequently, the latter group was divided into the NERD-SI+ group (with positive symptom index) and NERD-SI- group (with negative symptom index). The symptom index (SI) was defined as the number of times a symptom occurs in association with acid reflux (pH <4) within a 5-minute time period divided by the total number of times the symptom occurs [32], and SI ≥50% is considered positive. In the NERD-SI- group, the patients were asked to undergo a PPI trial (omeprazole 20 mg twice daily for 7 days), and those whose RDQ scores showed over 50% improvement were placed in the NERD-SI-group. If the score did not show 50% improvement, the subject was excluded from this study [7]. The NERD-SI- patients were evaluated with the fMRI study sequence at least two weeks after the PPI trial.

### Psychological states assessment

The Symptom Check List-90 was used for psychological assessment. It is a self-reported symptom inventory reflecting the psychological distress of an individual, which includes 90 items that cover nine psychological areas such as somatization, obsessive-compulsive disorder, interpersonal relationship sensitivity, depression, anxiety, hostility, phobic anxiety, paranoid ideation and psychoticism. It is interpreted using 9 primary symptom types and 3 global indices of distress. Higher scores indicate more distress.

### Assessment of cerebral responses to stimuli

#### MRI scanning

MR imaging was performed with a 1.5T Magnetom Sonata MR scanner (Siemens Medical Solutions, Erlangen, Germany) with a standard CP-array head coil. Twenty five axial T1-weighted images covering the whole brain were first acquired with a turbo spin echo (TSE) sequence. The scan parameters were as follows: repetition time (TR) = 450 ms, echo time (TE) = 7.7 ms, flip angle (FA) = 90°, field of view (FOV) = 220 × 220 mm, matrix = 256 × 256, slice thickness (ST) = 5 mm, slice gap = 1 mm. Next, high-resolution 3 D T1-weighted images were acquired with magnetization prepared for rapid gradient echo imaging (MPRAGE) with the following parameters: TR = 1900 ms, TE = 3.93 ms, FA = 15°, FOV = 220 × 220 mm, matrix = 256 × 256. ST = 1.7 mm, slice gap = 0.9 mm; a total of 96 slices were evaluated. Finally, a blood oxygen level dependent (BOLD) sensitive gradient echo, single shot echo planar imaging (GRE-EPI) sequence was used for fMRI data acquisition with the following parameters: TR = 6,000 ms, TE = 40 ms, FA = 90°, FOV = 220 × 220 mm, matrix = 64 × 64, ST = 5 mm, slice gap = 1 mm. In each of 25 contiguous slices 350 images were captured.

#### Intraesophageal acid stimuli and psychological anticipation protocol

**Figure 1:**
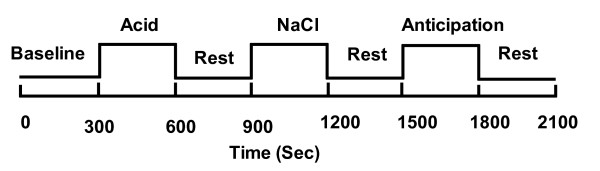
**Block design model and time course of fMRI signals for a responsive brain voxel**. Baseline: period prior to any stimulus. Acid: acid infusion in the esophagus. Rest: period without any stimulus. NaCl: isotonic saline infusion in the esophagus. Anticipation: induction of psychological anticipation stimulation.

A block design was used for fMRI scanning. The scanning lasted for 35-minutes, and this interval was segmented into seven equal 5-minute periods: rest 1, intraesophageal acid infusion, rest 2, intraesophageal isotonic saline infusion, rest 3, psychological anticipation stimuli and rest 4 (Figure 1). A multi-lumen catheter with four lateral infusion holes (situated at 1 cm intervals and each at a 90° angle) was inserted trans-nasally. The distal hole of the catheter was positioned 5 cm above the upper margin of the LES. For esophageal acid stimulation, room temperature 0.1N HCL was infused into the esophagus at a rate of 10 ml/min for 5 minutes, and study participants were not told the infusion was going on. Intraesophageal isotonic saline infusion (at a rate of 10 ml/min) was used to dilute and wash away the effects of the acid. At the beginning of the psychological anticipation test, the participant was informed through an earphone that an acid solution which might cause heartburn would be infused into his/her esophagus in the following 5 minutes. Then room temperature isotonic saline (with no acid) was infused into the esophagus for 5 minutes and the participant was informed when the infusion was completed by a stop signal.

#### Data analysis

The fMRI data were analyzed and can be found for review at the site Analysis of Functional NeuroImaging (AFNI) http://afni.nimh.nih.gov/afni. The first six images in the scan sequence were discarded to rule out nonequilibrium effects of magnetization and the remaining 350 functional images were divided into two parts for study analysis including an acid stimulation interval (1 TR to 150 TR) and an psychological stimulation interval (201 TR to 350 TR). Head motion correction and edge detection algorithms were first applied to the functional data. Functional activations were then generated by group t test analysis. Based on activation maps and previous literature, three ROIs including the ACC, PFC and IC were chosen for further statistical analysis. To confirm the reliability of the above analysis, fMRI signals were modeled after the beta distribution by using a nonlinear regression technique [33]. The beta distribution was chosen on empirical grounds. The onset and peak time of the beta model was constrained to occur within 3.0 and 6.6 minutes respectively after stimulation onset [15]. Other parameters of the beta distribution were loosely constrained in order that a best-fit model for each voxel's time series could be attained. We used onset-time [the time from rest 1 (1st TR) to the onset of fMRI signal response during stimulation], peak-time [the time from rest 1 (1st TR) to the peak of fMRI signal response curve during stimulation], offset-time [the time from rest 1 (1st TR) to the time of fMRI signal return to baseline during stimulation] and MAX% [the maximum signal change percent] to analyze the intragroup cerebral activation. Because the offset-times in some subjects were more than 150 TR, we employed 50% of the offset-time for analysis. We also analyzed inter-group differences in cerebral activation, as well as the differences in activation in actual esophageal acid perfusion and induced psychological anticipation with saline only infusion.

### Statistic Analysis

Statistic comparisons were performed using SPSS 11.0. Data are shown as mean ± SE and skew data is shown as quartile values. One-way ANOVA was used to analyze the differences among groups based on age, DeMeester scores, psychological measures and cerebral response data. Because of the small sample size in each group, we used Bonferroni adjustments to reduce the risk of type-I error. The MannWhitney U test was used to test the skew data. The mean difference is significant at the 0.05 level.

## Results

### Demographic and clinical data

GERD patients were divided into four subgroups as RE, NERD+, NERD-SI+ and NERD-SI-. The average age of RE and NERD-SI+ patients was higher than that of the controls (*P *< 0.05). GERD subgroups showed no statistic difference in GER symptoms. RE and NERD+ patients had more esophageal acid exposure than NERD-SI+, NERD-SI- and control groups, but there was no statistical difference in acid exposure in RE and NERD+ patients (Table 1).

**Table 1:** Subjects Demographics and Clinical Characteristics among Subgroups of GERD Patients and Controls

		RE N = 9	NERD+ N = 11	NERD-SI+ N = 8	NERD-SI- N = 11	Control N = 11
**Demographic characteristics**	Age, yrs (Mean ± SE)	56.7 ± 5.9	44.5 ± 3.9	58.1 ± 3.8	47.9 ± 2.2	38.0 ± 3.7 ^a^
	Sex (M/F)	7/2	6/5	4/4	5/6	5/6
**Symptom assessment** **In RDQ**	Frequency (Mean ± SE)	7.9 ± 2.8	9.5 ± 2.4	7.9 ± 1.1	9.0 ± 1.5	-
	Extent (Mean ± SE)	7.7 ± 3.0	9.5 ± 2.4	7.8 ± 1.4	9.2 ± 1.8	-
	Total score (Mean ± SE)	15.6 ± 5.7	19.1 ± 4.2	15.6 ± 2.2	18.2 ± 3.0	-
**24 hr pH monitoring**	SI [M (QR)]	100 (0-100)	60 (16.7-100)	100 (91.67-100)	0 (0)	-
	DM scores [M (QR)]	28.6 (13.9-51.4) ^b^	37.4 (27.7-90.6) ^c^	6.9 (3.6-12.6)	3.9 (1.0-7.1)	5.8 (4.6-9.6)

a: vs.RE, *P *= 0.015; vs. NERD-SI+, *P *= 0.011;

b: vs. NERD-SI+, NERD-SI- and Control, *P *< 0.001, respectively

c: vs. NERD-SI+, NERD-SI- and Control, *P *< 0.001, respectively

### Differences of the psychological states among the groups

Table 2 shows the scores for the SCL-90 questionnaire. All GERD subgroups had higher scores for most items than controls, but after Bonferroni adjustment only the "positive items" and somatization scores showed significant differences (*P *= 0.10~0.000). NERD-SI- subjects showed the highest scores among the GERD subgroups, but this was not statistically significant.

**Table 2:** Comparison of the Mean ± SE SCL-90 Scores among Subgroups of GERD Patients and controls

	Control (a) N = 11	RE (b) N = 9	NERD+ (c) N = 11	NERD-SI+ (d) N = 8	NERD-SI- (e) N = 11	ANOVA		
						
						F^Δ^	P*	Post Hoc^#^
**Total scores**	14.82 ± 5.67	27.78 ± 4.54	37.09 ± 7.33	32.75 ± 12.14	47.55 ± 11.72	2.08	0.099	-
**General symptomatic index**	0.16 ± 0.06	0.31 ± 0.05	0.41 ± 0.08	0.36 ± 0.13	0.53 ± 0.13	2.08	0.099	-
**Positive Items**	13.18 ± 4.89	20.33 ± 3.26	25.64 ± 4.15	19.38 ± 5.06	30.82 ± 6.53	7.18	0.000	a/b,a/c,a/d,a/e
**Obsessive-compulsive**	0.33 ± 0.11	0.57 ± 0.09	0.48 ± 0.11	0.56 ± 0.27	0.70 ± 0.18	0.83	0.511	-
**Somatization**	0.12 ± 0.05	0.45 ± 0.09	0.59 ± 0.12	0.63 ± 0.17	0.48 ± 0.06	3.80	0.010	a/c,a/d,a/e
**Anxiety**	0.14 ± 0.07	0.20 ± 0.09	0.28 ± 0.11	0.31 ± 0.19	0.45 ± 0.12	1.08	0.377	-
**Depression**	0.15 ± 0.06	0.37 ± 0.09	0.33 ± 0.08	0.47 ± 0.15	0.52 ± 0.16	1.63	0.183	-
**Interpersonal sensitivity**	0.13 ± 0.05	0.20 ± 0.06	0.34 ± 0.12	0.13 ± 0.05	0.52 ± 0.20	1.94	0.121	-
**Hostility**	0.15 ± 0.07	0.26 ± 0.10	0.48 ± 0.11	0.10 ± 0.04	0.56 ± 0.19	2.61	0.048	-
**Psychoticism**	0.10 ± 0.05	0.17 ± 0.05	0.26 ± 0.06	0.21 ± 0.10	0.37 ± 0.14	1.37	0.259	-
**Paranoid ideation**	0.17 ± 0.07	0.19 ± 0.10	0.33 ± 0.10	0.08 ± 0.04	0.35 ± 0.16	0.98	0.430	-
**Phobic anxiety**	0.16 ± 0.07	0.03 ± 0.02	0.19 ± 0.12	0.20 ± 0.13	0.21 ± 0.04	0.51	0.726	-

^Δ ^F values of One-Way ANOVA.

* *P *values of One-Way ANOVA.

^# ^Post hoc significant differences between the groups after Bonferroni adjustment

### Cerebral responses to intraesophageal acid stimulation

**Figure 2:**
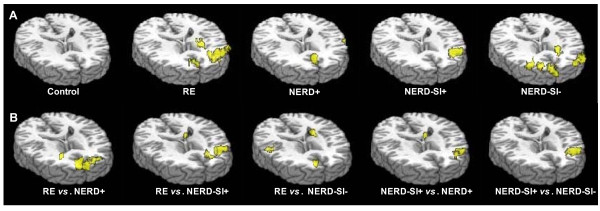
**Characteristic brain activation in the four GERD subgroups and controls**. 2A. Average intra-group cerebral activation during esophageal acid infusion: Control group: no ROI was activated. RE group: PFC, IC and ACC were activated. NERD+ group: PFC and IC were activated. NERD-SI+ group: PFC was activated. NERD-SI- group: PFC, IC and ACC were activated. 2B. Average inter-group cerebral activation during esophageal acid infusion. RE vs. NERD+: PFC and ACC were significantly activated in RE compared with the NERD group. RE vs. NERD-SI+: ACC and IC were significantly activated in RE compared with the NERD-SI+ group. RE vs. NERD-SI-: PFC and IC were significantly activated in RE compared with the NERD-SI- group. NERD-SI+ vs. NERD+: PFC were significantly activated in NERD-SI+ compared with the NERD+ group. NERD-SI+ vs. NERD-SI-: The PFC was significantly activated in NERD-SI+ compared with the NERD-SI- group.

**Figure 3:**
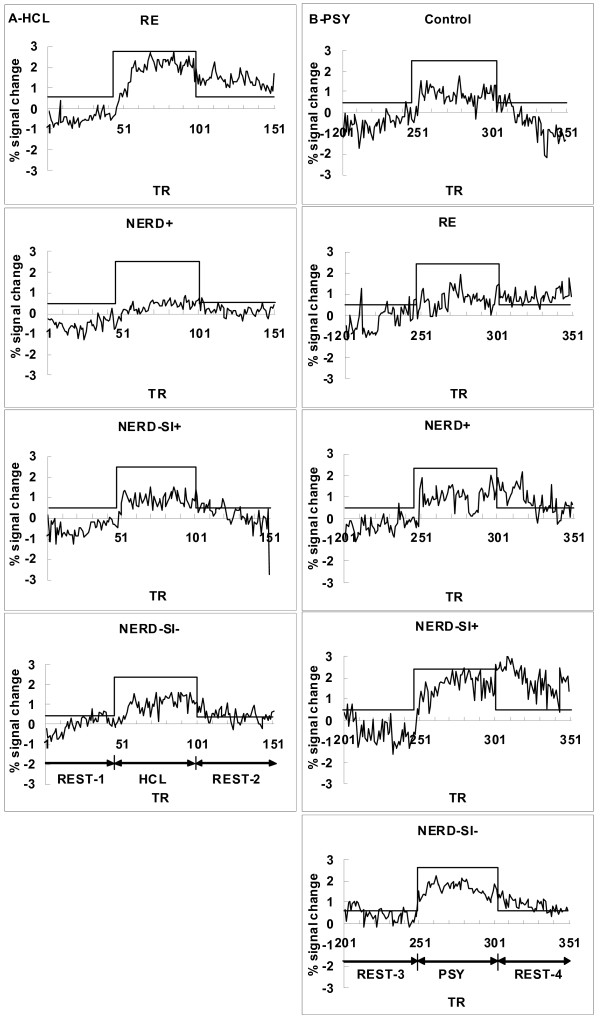
**Average fMRI signal change in GERD patients and controls**. 3A: Response to intraesophageal acid infusion in four GERD subgroups. The increase in fMRI magnetic signal intensity (MAX%) was greatest in the RE group in both the PFC and ACC. 3B: Response to induced psychological anticipation in the control group and the four GERD subgroups. The NERD-SI- group showed a shorter onset time than NERD+ group in PFC. NERD-SI- had significantly shorter peak times than RE group.

**Figure 4:**
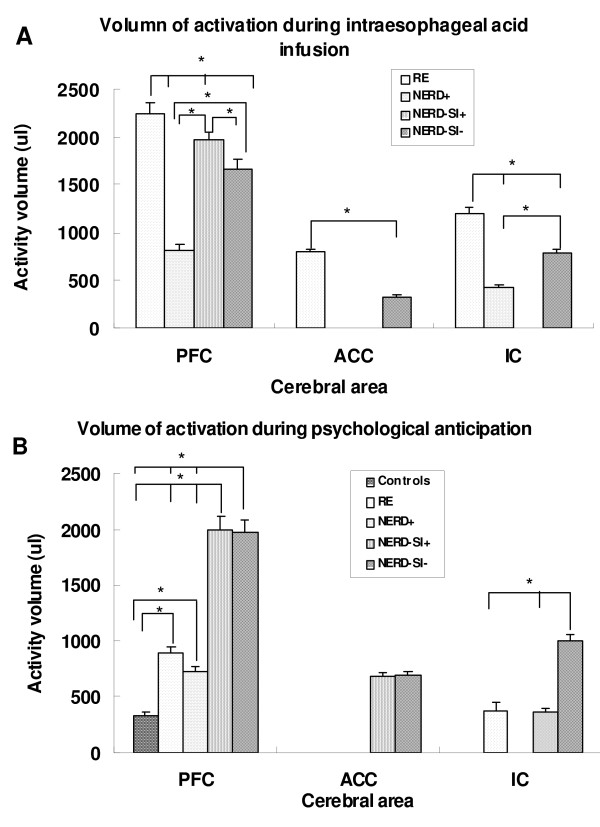
**Differences of activation volumes among the four GERD subgroups**. 4A: Activity volume during intraesophageal acid infusion RE group presented higher activity volume in PFC, ACC and IC than any other GERD group (p < 0.05). NERD-SI+ group presented higher activity volume in PFC than NERD-SI- and NERD+ groups (p < 0.05). NERD-SI- groups presented higher activity volume in PFC and IC than that of NERD+ group (p < 0.05). 4B: Activity volume during psychological anticipation NERD-SI+ and NERD-SI- presented higher activity volume in PFC than any other GERD groups (p < 0.05). RE and NERD+ group presented higher activity volume in PFC than control. NERD-SI- presented higher activity volume in IC than NERD+ and NERD-SI+.

Four GERD patients (2 RE, 1 NERD+ and 1 NERD-SI-) reported heartburn during acid infusion and the symptom was eased by saline infusion, but no other subject (all controls and other GERD patients) reported any discomfort during acid infusion. Regarding BOLD signals, there was no significant cerebral activation in the controls during intraesophageal acid perfusion. In contrast, the PFC region was significantly activated in all subtypes of GERD. In addition, the ACC regions were activated in RE and NERD-SI-groups but not in NERD+ and NERD-SI+ patients. The IC regions were activated in RE, NERD+ and NERD-SI-groups but not in NERD-SI+ patients (Figure 2A, Table 3). Table 4 and Figure 3A show the temporal and signal intensity characteristics and Figure 4A shows the activity volume of BOLD responses in the PFC, IC and ACC regions to intraesophageal acid stimulation in the GERD patients. The RE subgroup had the shortest peak-time and most extended volume in the PFC region after acid was infused, and the maximal fMRI signal intensity (MAX %) of this group was higher than that of the other three GERD subgroups. The activity volume of PFC in NERD-SI+ was more extended than that of NERD+ group and NERD-SI- group. In the ACC regions, the RE group also showed the highest MAX% and volume value, and these value were higher than that of the NERD-SI- group. The temporal characteristics showed no differences in the IC region among the RE, NERD+ and NERD-SI- groups.

**Table 3:** The Major Brain Region Activated in Subgroups of GERD Patients and Controls during Intraesophageal Acid and Psychological Anticipation Stimuli

Stimuli	ROI	RE					NERD+					NERD-SI+					NERD-SI-					Control				
		
		S	BA	x	y	z	S	BA	x	y	z	S	BA	x	y	z	S	BA	x	y	z	S	BA	x	y	z
**HCL**	PFC	B	BA11	-19	44	-27	L	BA11	-13	48	-31	L	BA11	-27	32	-26	L	BA11	-21	48	-27	-	-	-	-	-
	IC	L	BA13	-30	20	-2	L	BA13	-39	7	9	-	-	-	-	-	L	BA13	-37	9	-2	-	-	-	-	-
	ACC	R	BA32	4	44	0	L					-	-	-	-	-	R	BA25	3	20	0	-	-	-	-	-

**PSY**	PFC	B	BA11	-2	44	-27	L	BA11	-7	48	-20	B	BA11	-10	45	-27	B	BA11	-2	43	-27	R	BA11	-3	36	-24
	IC	L	BA13	-38	6	-8	-	-	-	-	-	R	BA13	38	7	8	L	BA13	-37	5	9	-	-	-	-	-
	ACC	-	-	-	-	-	-	-	-	-	-	B	BA24	8	-34	5	B	BA25	7	-33	8	-	-	-	-	-

HCL: Intraesophageal acid infusion; PSY: psychological anticipation stimulation

ROI: Regions of interesting

BA: Brodmann area

S: side

L: Left side

R: Right side

B: Bi-side

*x, y, z*: Coordinate in Talaraich

**Table 4:** Comparison of the Temporal Characteristics in PFC, IC and ACC among Subgroups of GERD Patients during Intraesophageal Acid and Psychological Anticipation Stimuli (Mean ± SE)

	ROI	Parameters	Control	RE (b)	NERD+ (c)	NERD-SI+ (d)	NERD-SI- (e)	ANOVA		
								
								F (t)^Δ^	P*	Post Hoc^#^
**HCL**	**PFC**	Onset t (TR)	-	60.8 ± 2.7	66.6 ± 3.2	57.4 ± 0.9	60.3 ± 1.3	2.65	0.074	
		Peak t (TR)	-	85.7 ± 3.0	93.7 ± 3.5	91.8 ± 1.8	93.8 ± 1.4	2.12	0.126	
		50%Offset t (TR)	-	109.8 ± 4.8	119.9 ± 3.5	123.6 ± 3.6	122.8 ± 3.5	2.48	0.088	
		MAX (%)	-	2.7 ± 0.2	1.3 ± 0.3	1.6 ± 0.3	1.7 ± 0.2	5.05	0.008	b/c
	**ACC**	Onset t (TR)	-	63 ± 2	-	-	60 ± 3	(0.60)	0.565	
		Peak t (TR)	-	88.3 ± 2	-	-	91 ± 3	(0.74)	0.481	
		50%Offset t (TR)	-	117 ± 3	-	-	119 ± 4	(0.37)	0.722	
		MAX (%)	-	4.2 ± 0.5	-	-	1.6 ± 0.1	(5.09)	0.001	b/e
	**IC**	Onset t (TR)	-	57 ± 3	56 ± 1	-	62 ± 3	0.64	0.546	
		Peak t (TR)	-	88 ± 2	90 ± 4	-	95 ± 4	0.82	0.464	
		50%Offset t (TR)	-	125 ± 2	138 ± 4	-	122 ± 6	1.02	0.391	
		MAX (%)	-	1.9 ± 0.1	1.7 ± 0.3	-	2.0 ± 0.3	0.34	0.720	

**PSY**	**PFC**	Onset t (TR)	57 ± 2.2	56.3 ± 1.5	58.5 ± 3.0	52.3 ± 1.0	50.8 ± 0.4	4.72	0.008	e/c
		Peak t (TR)	83 ± 2.6	83.3 ± 2.0	105.0 ± 7.9	99.3 ± 7.2	70.8 ± 2.4	10.88	0.000	e/c,e/d
		50%Offset t (TR)	117.6 ± 4.2	101.2 ± 3.6	126.5 ± 7.1	130.0 ± 10.7	126.1 ± 9.5	1.51	0.237	
		MAX (%)	1.7 ± 0.1	1.9 ± 0.2	2.2 ± 0.2	3.0 ± 0.3	2.5 ± 0.3	2.59	0.068	
	**ACC**	Onset t (TR)	-	-	-	54 ± 1	52 ± 1	(0.96)	0.360	
		Peak t (TR)	-	-	-	85 ± 5	80 ± 3	(0.96)	0.360	
		50%Offset t (TR)	-	-	-	117 ± 6	126 ± 7	(0.89)	0.394	
		MAX (%)	-	-	-	2.7 ± 0.4	1.9 ± 0.4	(1.22)	0.250	
	**IC**	Onset t (TR)	-	54 ± 1	-	52 ± 1	52 ± 1	1.17	0.350	
		Peak t (TR)	-	85 ± 3	-	66 ± 2	78 ± 4	9.88	0.004	d/b
		50%Offset t (TR)	-	119 ± 3	-	115 ± 3	103 ± 2	5.21	0.028	e/b
		MAX (%)	-	1.9 ± 0.5	-	1.3 ± 0.1	2.0 ± 0.2	1.49	0.271	

HCL: Intraesophageal acid infusion; PSY: psychological anticipation stimulation; TR: repetition time

**^Δ ^**F values of One-Way ANOVA or t value of student t-test.

* *P *values of One-Way ANOVA or student t-test.

^# ^Post hoc significant differences between the groups after Bonferroni adjustment.

### Cerebral response to psychological anticipation stimulation

**Figure 5:**
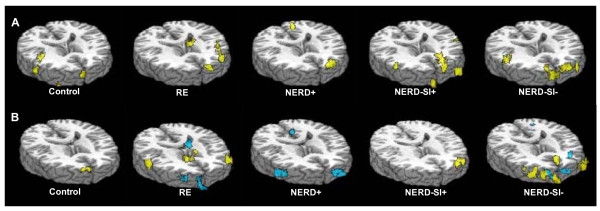
**Intragroup brain activation differences during induction of psychological anticipation among GERD subgroups and control**. 5A. Average intragroup cerebral activation during induction of psychological anticipation: Control group: PFC was activated. RE group: PFC and IC were activated. NERD+ group: PFC was activated. NERD-SI+ group: PFC, IC and ACC were activated. NERD-SI- group: PFC, IC and ACC were activated. 5B. Comparing psychological anticipation with actual acid infusion, based on cerebral activation (yellow-colored) and deactivation (blue-colored) in the four GERD subgroups Control: PFC was activated in psychological anticipation when compared with actual acid infusion. RE group: ACC was deactivated. The intensity of PFC in anticipation was lower than that of actual acid infusion. NERD+ group: IC was deactivated. The intensity of PFC in anticipation was lower than that of actual acid infusion NERD-SI+ group: PFC was activated more extensively during anticipation than acid infusion. NERD-SI- group: The intensity of activation of most of the PFC area and ACC in anticipation was higher, and a small part of the PFC was lower than that of actual acid infusion.

During induced psychological anticipation stimulation, the PFC was significantly activated both in the control and GERD groups. Activation of the IC region was found in the RE, NERD-SI+ and NERD-SI- subgroups but not in the NERD+ group. Only NERD-SI+ and NERD-SI- patients showed ACC activation (Figure 5A). In the PFC region, the NERD-SI- subgroup had the shortest onset time and peak-time. The activity volumes of NERD-SI+ and NERD-SI- were more extended than those of controls and other GERD groups. Moreover, as compared with the RE subgroup and the controls, all NERD subjects had longer 50% offset-time, although the differences were not statistically significant after Bonferroni adjustment. In the IC region, the NERD-SI+ group showed the shortest peak-time. The temporal characteristics of the ACC response showed no difference in the NERD-SI+ and NERD-SI- groups (Figure 3B, 4B; Table 3, 4).

### Differences in cerebral activation in actual esophageal acid perfusion and psychological anticipation

fMRI signal activation differences in actual esophageal acid perfusion and induced psychological anticipation stimuli were assessed in each GERD subtype. All five groups showed cerebral activation during the anticipation step, and multiple differences in activation area and temporal characteristics emerged. During anticipation in the RE group, the ACC was deactivated while the PFC was activated, but the signal intensity of the PFC was significantly lower in under induced anticipation than in actual acid perfusion stimulation (p = 0.010). In the NERD+ group, the IC was deactivated during anticipation. But there was no significant difference in the PFC activation in NERD+. In contrast with the RE and NERD+ groups, more cerebral areas including the IC (BA 13) and the ACC (BA24) were activated in the NERD-SI+ group during psychological anticipation. In the NERD-SI- group, although the activated area did not show much change, the onset-time and peak-time in the PFC and IC areas were obviously shorter in anticipation stimulation than actual acid infusion (p < 0.001, p < 0.001, p = 0.038, p = 0.038, respectively). The onset-time in the ACC was also shorter with induced anticipation (p = 0.030) (Figure 5B).

## Discussion

In the current study, we analyzed characteristics of visceral hypersensitivity among four subgroups of GERD, including RE, NERD+, NERD-SI+ and NERD-SI- groups and assessed the different cerebral effects of induced psychological anticipation stimulation on these four groups as compared with controls. During intraesophageal acid stimulation, we found GERD patients showed cerebral activation, with activation of the PFC in all four subgroups, and activation of the ACC, the IC in three subgroups (RE, NERD+, NERD-SI-). However, no ROIs were activated in the controls. These ROIs have been shown to be related to esophageal perception and sensation in GERD patients during esophageal acid perfusion or balloon distention in previous studies [15,27]. Consistent with what has been previously reported, GERD patients showed central hypersensitivity to esophageal acid perfusion as compared with controls [15]. In this study, an HCL perfusion rate of 10 ml/min 0.1 N was chosen according to the Bernstein test. Several studies have reported this perfusion rate would not induce symptoms in controls within 10 minutes [34]. However, when the test time was extended to 20 minutes, three of 15 controls reported chest discomfort during acid perfusion [35]. To ensure there would be no detectable "harmful" response, and to exclude head motion during a long scan time, we designed this study to employ a shorter length of time for acid infusion (5 minutes) and confirmed there was no significant cerebral activation in healthy controls within this time period. GERD patients also showed a different psychological profile with higher mental test scores than controls, especially in the "positive items" and somatization. This result was similar to that in the study by Baker et al. They found the average scores for somatization, anxiety and depression were higher in 51 GERD patients than in 43 controls [36].

### Four subtypes of GERD presented different cerebral activation patterns associated with intraesophageal acid perfusion

The RE group showed the most extensive activated areas (PFC, ACC and IC) and took a shorter time to reach signal activation peak in PFC during esophageal acid perfusion. Moreover, the reactive intensity of magnetic signals (MAX% values in both PFC and ACC regions) and cerebral activity volume were highest in the RE group as compared with the other GERD subtypes, indicating that this group has the most pronounced hypersensitivity to intraesophageal acid stimulus among the four subtypes of GERD. Some studies have reported on visceral sensation in RE mainly by evaluating symptoms resulting from intraesophageal acid perfusion or balloon distention, but results have been conflicting [22,23]. In the current study, we analyzed cerebral activation and confirmed the RE group has much higher visceral sensitivity than NERD+, NERD-SI+ and NERD-SI-. The chronic tissue injury in the esophageal mucosa of RE patients may expose neural fibers to excessive acid, which may in turn enhance chemosensitivity [10]. Moreover, esophageal pain hypersensitivity to experimental acid infusion can be reversed by acid suppression via the proton pump inhibitors [13]. Long-term acid exposure may therefore be assumed to be the main mechanism for RE hypersensitivity.

The NERD+ group showed moderate ROI activation (PFC and IC) during esophageal perfusion. The average activation was more prominent in this group than in healthy controls, but no significant differences in activation time or signal changes were found in NERD+ versus other GERD subtypes. These findings are partly in agreement with previous observations by Marrero et al. who showed enhanced sensitivity to esophageal acid perfusion in patients with symptoms of GERD, and abnormal 24-hr esophageal pH monitoring studies, but no histological evidence of esophageal inflammation (NERD+ group) as compared with healthy controls [37].

NERD-SI+ showed the PFC activation with esophageal stimulation. When compared with NERD+, the extent of activation in the PFC was greater (p < 0.05) and the onset-time was shorter, although without significant difference. Fundamentally, positive symptom index means "acid-sensitive esophagus" [7], in the current study, we found the NERD-SI+ group showed more esophageal sensitively than the controls and NERD+ group.

In the NERD-SI- group, the three ROI areas were all activated and were more extensive as compared with activation in NERD+. In a recent study of NERD and functional heartburn patients, there was a correlation between pain threshold and acid exposure, and increased esophageal sensitivity was associated with a lower DeMeester score. Thus reflux negative (including NERD-SI- and functional heartburn) patients had lower pain thresholds as compared both with reflux positive patients and controls [38]. Our findings were consistent with this result.

In summary, during esophageal acid perfusion the four subgroups of GERD showed distinctly different activation patterns and all were hypersensitive as compared with controls. Among these groups, the RE group showed the most obvious acid sensitivity.

### Psychological anticipation of intraesophageal acid perfusion caused different cerebral activation patterns among the four subtypes of GERD

Under induced psychological anticipation, all of the GERD subgroups and controls developed cerebral activation. This result is partly in accordance with the study by Yaguez et al. which showed that anticipated visceral pain elicited cortical responses in healthy subjects [29]. These findings imply that the cerebral perception and sensation of visceral stimuli can be modulated by psychological factors. In fact, there have been increasing numbers of reports which support the view that emotional states, in particular anxiety and anger, are closely associated with greater perceptual and physiologic responses to visceral stimuli. Wright et al revealed that the perception of symptoms is increased when the NERD- patients are stressed [39]. Fass et al also demonstrated that acute auditory stress can exacerbate heartburn symptoms in GERD patients by enhancing perceptual response to intraesophageal acid exposure, which is associated with greater emotional responses to the stressor [21]. Sabate et al suggested anxiety and coping were significantly related with IBS patients' pain thresholds [40]. Some studies have shown that limbic areas in healthy volunteers and rheumatic arthritis patients, such as the ACC and medial PFC are associated with pain-related anxiety or depression during pain processing [41-43]. A few studies have addressed anticipation of visceral stimulation. Berman et al reported reduced brainstem inhibition during anticipated pelvic visceral pain correlates with enhanced brain response to the visceral stimulus in women with IBS [30]. Yaguez et al reported actual and anticipated esophageal balloon distention could elicit similar cortical responses [29]. In our study, analysis showed there were different patterns among four groups of patients. RE showed deactivation of the ACC and NERD+ showed the IC area deactivated, while NERD-SI+ showed a greater activation in the ACC and the IC and NERD-SI- showed shorter onset-time and peak-time for activation in the PFC and the IC during anticipation. These results suggest psychological factors play a more important role in NERD-SI+ and NERD-SI- groups than in RE and NERD+.

NERD and RE are generally considered to represent a spectrum of GERD which ranges from mild (NERD) to severe (RE) [44]. To the best of our knowledge, this is the first study to demonstrate that subtypes of NERD, including NERD+, NERD-SI+ and NERD-SI-, display heterogeneous perception and visceral sensation using fMRI. During esophageal acid stimulation, the four subgroups of GERD presented different activation patterns and all were hypersensitive as compared with controls. Among these groups, RE showed the most obvious acid sensitivity. The four subgroups also showed different patterns under induced psychological anticipation, and psychological factors play a more important role in NERD-SI+ and NERD-SI- groups. This likely corresponds to the fact that patients in these two subgroups respond to PPI therapy poorly, and have important implications for improved understanding of the pathogenesis of the four subgroups of GERD, and particularly of the NERD subgroups.

There were some limitations in the current study. First, the mean ages of subjects in our study were not matched precisely among the subgroups and controls. Studies regarding the relationship between age and cerebral activation suggest that older control subjects show significantly less activation in PFC than younger controls [45]. In GERD, older patients showed reduced esophageal chemosensitivity to acid as compared to younger patients [46]. In our study, when age mismatch was taken into consideration, the GERD patients were even more sensitive to intraesophageal acid stimulation and psychological anticipation than controls. The second limitation of the current study is that the PPI responses in the NERD groups (except for the NERD-SI- group) were not checked. However, we enrolled these NERD patients according to the results of RDQ symptom questionnaire, gastroscopy and 24-h pH monitoring and none of these patients had PPI therapy prior to testing. The effectiveness of PPI on visceral sensation and perception might be an aspect to evaluate further.

## Conclusions

The four subgroups of GERD patients and controls showed distinctly different activation patterns and we therefore conclude GERD patients have different patterns of visceral perception and psychological anticipation. Psychological factors play a more important role in NERD-SI+ and NERD-SI- groups than in RE and NERD+ groups.

## List of abbreviations

GERD: Gastroesophageal reflux disease; RE: Reflux esophagitis; NERD: Non-erosive reflux disease; ACC: Anterior cingulated cortex; PFC: prefrontal cortex; IC: Insular cortex.

## Competing interests

The authors declare that they have no competing interests.

## Authors' contributions

LPD, Study concept and design; critical revision of the manuscript for content; obtained funding for the study; KW, Acquisition of data; statistical analysis and interpretation of data; drafting of the manuscript; XZZ, fMRI scanning and technical assistance, in acquisition and analysis of fMRI data; JYL, Magnetom Sonata MR scanner technical support, analysis and interpretation of fMRI data; XCW, Revision of the manuscript, instruction concerning interpretation of fMRI data; also obtained funding for the study.

All the authors have read and approved the final manuscript.

## Pre-publication history

The pre-publication history for this paper can be accessed here:

http://www.biomedcentral.com/1471-230X/11/28/prepub
